# Super‐Low‐Dose Functional and Molecular Photoacoustic Microscopy

**DOI:** 10.1002/advs.202302486

**Published:** 2023-06-13

**Authors:** Yachao Zhang, Jiangbo Chen, Jie Zhang, Jingyi Zhu, Chao Liu, Hongyan Sun, Lidai Wang

**Affiliations:** ^1^ Department of Biomedical Engineering City University of Hong Kong Hong Kong SAR 999077 China; ^2^ Department of Chemistry and COSADAF (Centre of Super‐Diamond and Advanced Films) City University of Hong Kong Hong Kong SAR 999077 China; ^3^ City University of Hong Kong Shenzhen Research Institute Shenzhen China 518057

**Keywords:** high sensitivity, deoxyhemoglobin imaging, molecular imaging, super low doses, low phototoxicity

## Abstract

Photoacoustic microscopy can image many biological molecules and nano‐agents in vivo via low‐scattering ultrasonic sensing. Insufficient sensitivity is a long‐standing obstacle for imaging low‐absorbing chromophores with less photobleaching or toxicity, reduced perturbation to delicate organs, and more choices of low‐power lasers. Here, the photoacoustic probe design is collaboratively optimized and a spectral‐spatial filter is implemented. A multi‐spectral super‐low‐dose photoacoustic microscopy (SLD‐PAM) is presented that improves the sensitivity by ≈33 times. SLD‐PAM can visualize microvessels and quantify oxygen saturation in vivo with ≈1% of the maximum permissible exposure, dramatically reducing potential phototoxicity or perturbation to normal tissue function, especially in imaging of delicate tissues, such as the eye and the brain. Capitalizing on the high sensitivity, direct imaging of deoxyhemoglobin concentration is achieved without spectral unmixing, avoiding wavelength‐dependent errors and computational noises. With reduced laser power, SLD‐PAM can reduce photobleaching by ≈85%. It is also demonstrated that SLD‐PAM achieves similar molecular imaging quality using 80% fewer contrast agents. Therefore, SLD‐PAM enables the use of a broader range of low‐absorbing nano‐agents, small molecules, and genetically encoded biomarkers, as well as more types of low‐power light sources in wide spectra. It is believed that SLD‐PAM offers a powerful tool for anatomical, functional, and molecular imaging.

## Graphical abstract

A multi‐spectral super‐low‐dose photoacoustic microscopy (SLD‐PAM) is presented. The SLD‐PAM realizes in vivo anatomical and functional label‐free imaging with only 1‐nanojoule pulse energy and implements direct imaging of deoxyhemoglobin concentration without spectral unmixing. Without compromising imaging quality, SLD‐PAM can reduce photobleaching by lowering laser dose or reduce perturbation to normal tissue function by using fewer contrast agents.

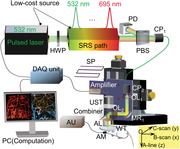

## 1 Introduction

Optical‐resolution photoacoustic microscopy (OR‐PAM) detects rich optical absorption contrasts at a sub‐cellular resolution ^[^ ^1^, ^2^, ^3^, ^4^, ^5^, ^6^, ^7^, ^8^ ^]^ and is being developed for studying a broad range of diseases, such as cancers, neurodegenerative diseases, diabetes, ocular diseases, cardiovascular disease, and strokes.^[^ ^9^, ^10^, ^11^, ^12^, ^13^, ^14^, ^15^ ^]^ High sensitivity is not only important for high‐quality imaging, but also can detect low‐absorbing low‐concentration chromophores, reduce photobleaching, phototoxicity, or perturbation, and broaden the choices of low‐cost low‐power lasers in a wide spectrum. For instance, in ophthalmologic examination,^[^ ^16^ ^]^ low‐power laser is preferred for more safety and comfort; in high‐speed photoacoustic imaging, increased pulse repetition rates (PRR) limit the laser pulse energy,^[^ ^17^, ^18^, ^19^, ^20^ ^]^ wearable or portable OR‐PAM prefers lightweight low‐power lasers;^[^ ^21^ ^]^ long‐term monitoring of pharmacokinetics or hemodynamics requires low‐dose imaging to alleviate perturbation to tissue function.^[^ ^22^ ^]^

OR‐PAM usually uses piezoelectric transducers to detect photoacoustic waves,^[^ ^23^, ^24^, ^25^, ^26^, ^27^ ^]^ and the sensitivity is high but still insufficient for many preclinical and clinical applications. The tight acoustic focus has been explored to increase detection sensitivity. However, the ultrasonic collection efficiency has not been comprehensively optimized. In the past decade, novel optical ultrasound sensors ^[^ ^28^, ^29^, ^30^ ^]^ have been explored to improve detection sensitivity. Although optical acoustic sensors have many advantages such as broad bandwidths and area‐independent sensitivity, due to various practical challenges, their sensitivity has not yet superseded focused piezoelectric transducers in reported in vivo results.^[^ ^31^ ^]^

Another approach to increase sensitivity is denoising. Averaging or encoded excitation methods can increase signal‐to‐noise ratio but suffer from slow imaging speed and increased average power.^[^ ^32^, ^33^ ^]^ Many denoising methods, such as the Gaussian filter, Frangi filter, FIR (finite impulse response) filter, or filters based on discrete wavelet transform, have been used for OR‐PAM.^[^ ^34^ ^]^ Most of them filter signals in one or two dimensions. However, photoacoustic signals in the 3D space and at different optical wavelengths are all coherent. Thus, the existing methods use only partial coherent signals, resulting in limited improvement of the signal‐to‐noise ratio. Deep‐learning approaches can effectively reduce noise, but inadequate, slow, and system‐dependent training has posed great challenges to further increase the sensitivity.^[^ ^35^, ^36^, ^37^ ^]^

Here, via optimizing the photoacoustic probe and developing a 4D spectral‐spatial filter algorithm, we present super‐low‐dose photoacoustic microscopy (SLD‐PAM) for ultrasensitive functional and molecular imaging. In this work, we improve the overall optical/acoustic design to collect three times more photoacoustic signals than our previous technique.^[^ ^19^ ^]^ The alignment between the optical and acoustic foci is improved. The 4D spectral‐spatial filter algorithm is developed based on discrete wavelet transform. The innovation is the use of almost all possible coherent signals in the spectral and spatial dimensions to suppress noises and recover weak signals. The collaborative engineering in hardware and computation increases the sensitivity by 6∼33 folds (in vivo results) than our previous system and enables super low dose functional and molecular imaging. Using tens of times less pulse energy than the lowest reported in vivo results, SLD‐PAM is demonstrated to image the microvessels and calculate oxygen saturation in the skin, the eye, and the brain, reducing possible perturbation to normal tissue functions. Capitalizing on high sensitivity, we use a low‐power 675‐nm laser to directly image deoxyhemoglobin (Deoxy‐Hb) concentration in vivo, which avoids wavelength‐dependent errors in spectral unmixing. In molecular imaging, SLD‐PAM can use low laser power and effectively reduces photobleaching by ∼85%. Without compromising imaging quality, we can inject ∼5 times fewer molecular agents to reduce biotoxicity or metabolic burden.

## 2 Results

### 2.1 Design and Characterization of the SLD‐PAM System

**Figure 1:**
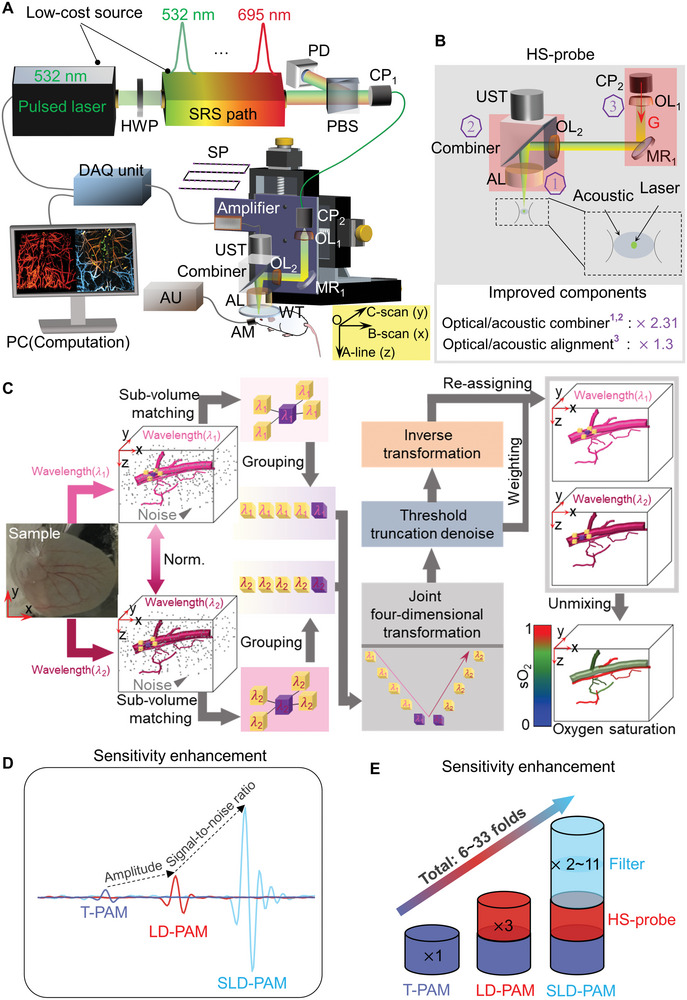
Schematic of multispectral SLD‐PAM and sensitivity enhancement. A) Schematic of the multispectral SLD‐PAM system. The SRS path composes of a low‐cost re‐configurable wavelength shifter. B) Schematic of the high‐sensitivity probe (HS‐probe). The numbers label the optimized design that improves detection sensitivity. C) Diagram of the spectral‐spatial filter algorithm. All coherent signals in neighboring 3D space and all‐optical wavelengths are joined to maximize the signal‐to‐noise ratio. D) Sensitivity comparison among traditional PAM (T‐PAM), PAM with high‐sensitivity probe only (LD‐PAM), and PAM with both high‐sensitivity probe and spectral‐spatial filter (SLD‐PAM). E) Sensitivity improvement from the probe and the filter. AL, acoustic lens; AM, anesthesia mask; AU, anesthesia unit; CP, coupler; DAQ, data acquisition; G, gravity; HWP, half‐wave plate; LD‐PAM, low‐dose photoacoustic microscopy; MR, mirror; OL, optical lens; PBS, polarizing beam splitter; PD, photodiode; SLD‐PAM, super‐low‐dose photoacoustic microscopy; SP, scanning pattern; SRS, stimulated Raman scattering; sO_2_, oxygen saturation; T‐PAM, traditional photoacoustic microscopy; UST, ultrasound transducer; WT, water tank.

As shown in **Figure** **1**, the SLD‐PAM system consists of a low‐cost multi‐wavelength laser (Figure 1A; Figure S1, Supporting Information), a high‐sensitivity probe (HS‐probe, Figure 1B; Figure S2, Supporting Information), a scanning and data acquisition module, and a 4D spectral‐spatial filter (Figure 1C). The laser is built with a 532‐nm pump laser and a fiber‐based stimulated Raman scattering (SRS) shifter. It can provide eleven wavelengths from 532 to 695 nm to image different chromophores. The HS‐probe delivers a focused laser beam to the tissue and collects photoacoustic waves. To increase sensitivity, the HS‐probe uses a high‐numerical‐aperture acoustic lens, optimizes the optical/acoustic beam combiner, and improves the optical/acoustic alignment. Although the acoustic lens has been used to increase sensitivity, our design approach the highest possible acoustic aperture. The optical/acoustic alignment is optimized via minimizing the gravity‐ and adhesive‐related deformation. As a result, acoustic waves can be collected and transmitted with higher efficiency than that in the original probe. The scanning and data acquisition module translates the probe and digitizes the signals. The spectral‐spatial filter denoises the three‐dimensional multi‐wavelength data to further improve the signal‐to‐noise ratio (SNR). Detailed descriptions of the laser source, HS‐probe, and spectral‐spatial filter are provided in Experimental Section.

We name photoacoustic microscopy with the HS‐probe only as low‐dose photoacoustic microscopy (LD‐PAM) and name the photoacoustic microscopy with both the HS‐probe and the spectral‐spatial filter as SLD‐PAM. LD‐PAM and SLD‐PAM share the same spatial resolution, and characterization of the spatial resolution and maximal imaging depth of the SLD‐PAM is in Supporting Information (see Figures S3 and S4, and Text S4, Supporting Information). Figure 1D,E shows representative sensitivity improvements by the HS‐probe and the spectral‐spatial filter. Note that the spectral‐spatial filter does not change the signal amplitude, but it suppresses the noise so that the signal‐to‐noise ratio is enhanced. The signals plotted in Figure 1D are normalized. The HS‐probe has approximately threefold sensitivity improvement compared with our previous system (see Experimental Section; Figure S2F, Supporting Information). We also compare the performance of LD‐PAM with traditional photoacoustic microscopy (T‐PAM) via in vivo microvessels imaging. The LD‐PAM outperforms the T‐PAM in 2D/3D imaging and provides more details under the same laser pulse energy (Figure S5, Supporting Information). The spectral‐spatial filter in the SLD‐PAM can further increase the signal‐to‐noise ratio by 2∼11 times (see Experimental Section; Figures S6–S8, Supporting Information). In total, SLD‐PAM is 6∼33‐folds more sensitive than traditional photoacoustic microscopy (T‐PAM) and requires super low‐dose excitation for in vivo functional and molecular imaging.

### 2.2 Photoacoustic Imaging of Oxygen Saturation and Morphology at Low Pulse Energy

**Figure 2:**
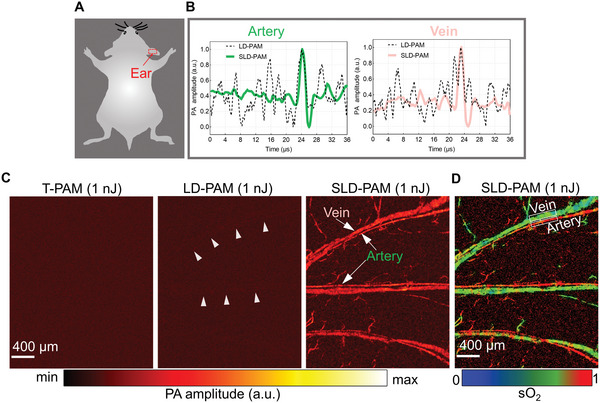
Comparison of in vivo results of T‐PAM, LD‐PAM, and SLD‐PAM at super low pulse energy. A) Illustration of the scanning region of the mouse. B) Comparison of representative A‐line signals acquired by LD‐PAM and SLD‐PAM without averaging. Laser pulse energy is 1 nJ, and the wavelength is 532 nm. C) Comparison of vascular imaging at 1‐nJ laser pulse energy acquired by T‐PAM, LD‐PAM, and SLD‐PAM. D) Oxygen saturation image at 1‐nJ laser pulse energy.

Enabled by high sensitivity, SLD‐PAM can image microvessels and oxygen saturation (sO_2_) in vivo using super low pulse energy. To demonstrate it, we use 532‐nm and 558‐nm wavelengths to image the mouse ear at various laser pulse energies. Representative A‐lines without and with the 4D spectral‐spatial filter are plotted in **Figure** **2**B. We can see that the filter can dramatically suppress the noise and enhance the visibility of weak signals. We compared microvascular images acquired with T‐PAM, LD‐PAM, and SLD‐PAM at 1‐nJ laser pulse energy (Figure 2C). Here, we use LD‐PAM images acquired at 64‐nJ laser pulse energy for reference (Figure S6, Supporting Information). The 1‐nJ excitation energy is less than 1% of the maximum permissible exposure (MPE, see text S5 in Supporting Information) on the tissue surface. At such low excitation power, T‐PAM cannot show any vessels. LD‐PAM barely distinguishes major vessels. SLD‐PAM can reveal major arteries and veins. More A‐line signals are shown in Movie S1 (Supporting Information). Figure S9 (Supporting Information) shows the super‐low‐dose group, covering the ear, the eye, and the brain imaging with laser energy of 0.5, 1, and 2 nJ. A full comparison of raw images and filtered results at the different wavelengths can be found in Figure S10 (Supporting Information). We also compare the 4D spectral‐spatial filter algorithm with other common denoising algorithms (Figure S11, Supporting Information), for example, average processing with dual‐wavelength, 3D Gaussian filter, the Frangi filter,^[^ ^38^ ^]^ and the wavelet filter.^[^ ^39^ ^]^ The proposed method has more advantages in noise suppression and signal enhancement.

As shown in Figure 2D, because the 4D spectral‐spatial filter maintains a linear relationship among different wavelengths, we can calculate sO_2_ from the low‐energy data. We compare the sO_2_ images acquired at different laser energies (Figure S12, Supporting Information). The 1‐nJ sO_2_ values in major vessels (65‐µm diameter for the vein and 25‐µm diameter for the artery) agree well with the 64‐nJ results. Compared with most reported vascular and sO_2_ imaging results that use several tens to hundreds of nanojoules of pulse energy, our results represent a great leap forward in super low‐dose functional imaging.

**Figure 3:**
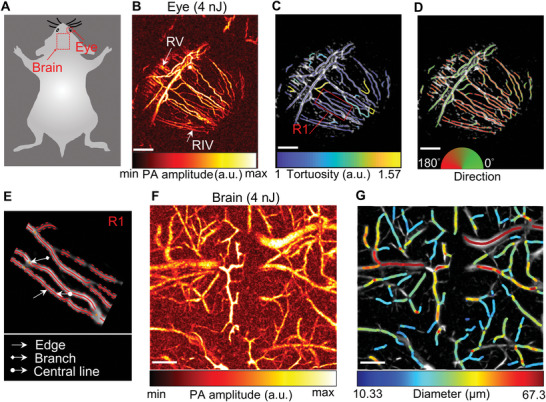
Vascular morphology extraction at super‐low‐dose illumination. A) Illustration of the regions of interest. B) Photoacoustic image of the ocular vasculature acquired at 4‐nJ pulse energy (532 nm). C) Tortuosity‐encoded and D) direction‐encoded photoacoustic image of the ocular vessels. E) Close‐up image in C) showing the edges, branches, and central lines of radial iris vessels. F) Photoacoustic image of the brain vasculature acquired at 4‐nJ pulse energy (532 nm) through the intact skull. G) Diameter‐encoded photoacoustic image of the brain vessels. RV, retinal vessel; RIV, radial iris vessel. Scale bars are 400 µm.

Microvascular morphology is of great importance for tissue regeneration or disease progressions, such as wound healing, inflammation, or tumor occurrence.^[^ ^40^, ^41^, ^42^ ^]^ Quantitation of vascular tortuosity, vascular direction, and diameter offers an important tool for disease diagnosis and monitoring. Vasculature segmentation, localization, and parameter extraction usually require high SNR. The state‐of‐the‐art OR‐PAM systems often use tens to hundreds of nanojoules of pulse energy, which may cause concerns of optical or heat perturbation to some delicate organs, like the eye or the brain cortex. Enabled by much improved SNR, SLD‐PAM can quantify vasculature morphology using only a few nanojoules. **Figure** **3** shows SLD‐PAM of the eye and the brain acquired with 4‐nJ pulse energy. We can easily localize the radial iris vessels and retinal vessels (Figure 3B) and quantify vascular tortuosity and direction (see Experimental Section; Figure 3C,D). Figure 3E shows extracted central lines, edges, and branches of radial iris vessels marked region in Figure 3C. Figure 3F is a photoacoustic image of the brain cortex with the intact cranium, where the vessel diameters range from 10.3 to 67.3 µm. Figure 3G is a diameter‐encoded photoacoustic image of the brain acquired with minimal perturbation to neural activities.

### 2.3 Deoxyhemoglobin Photoacoustic Microscopy (Deoxy‐Hb‐PAM)

Deoxy‐Hb concentration is highly related to tissue function. OR‐PAM can indirectly calculate Deoxy‐Hb concentration via spectral unmixing, but wavelength‐dependent optical attenuation may cause errors and computation may amplify noises. Thus, OR‐PAM often uses highly absorptive green light and high pulse energy to increase the SNR, causing nonlinear or potential phototoxicity problems. At microscopic resolution, Deoxy‐Hb has not been directly imaged in vivo due to the lack of proper laser and insensitive detection. In the red spectrum (634–676 nm), Deoxy‐Hb is nearly ten times more absorptive than Oxy‐Hb, thus, OR‐PAM can directly image Deoxy‐Hb concentration using a red wavelength without spectral unmixing. However, on the one hand, because the blood is one to two orders of magnitude less absorptive in the red spectrum than that in the green spectrum, OR‐PAM needs much higher laser pulse energy for in vivo imaging. On the other hand, pulsed lasers in the red spectrum have either inadequate pulse energy or low pulse repetition rate, making them not applicable for fast in vivo imaging.^[^ ^43^ ^]^

**Figure 4:**
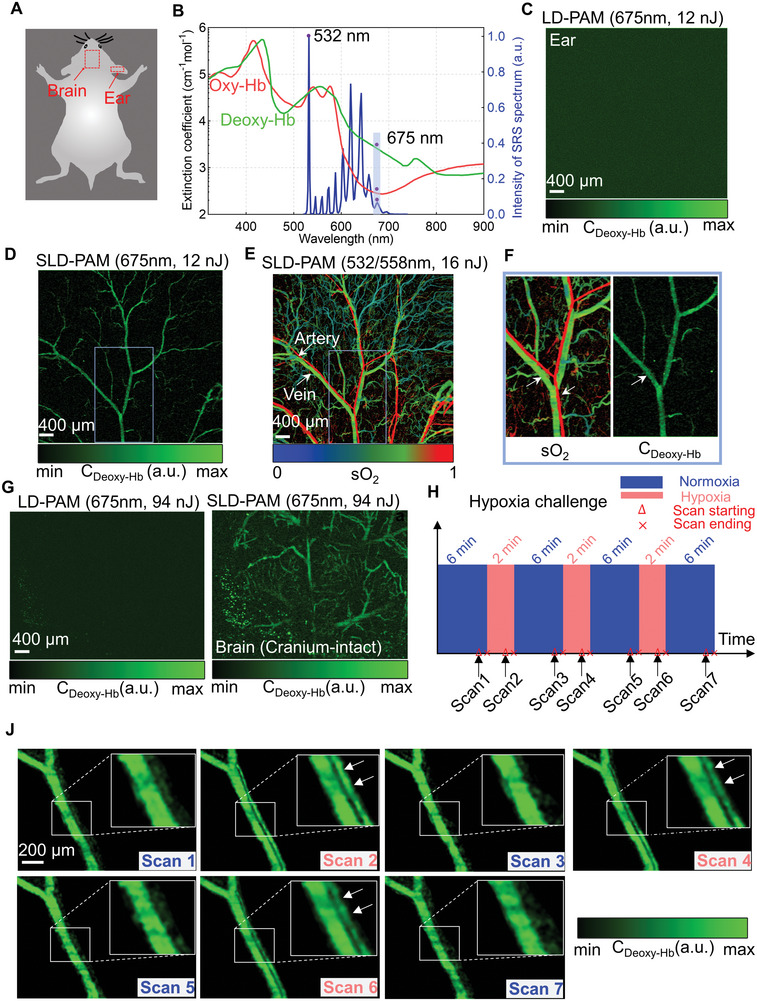
Deoxy‐Hb‐PAM at 675‐nm wavelength. A) Illustration of the regions of interest. B) Absorption spectra of oxyhemoglobin (Oxy‐Hb) and deoxyhemoglobin (Deoxy‐Hb), and spectrum of the low‐cost SRS pulsed laser. C) LD‐PAM and D) SLD‐PAM of the mouse ear at 675 nm with pulse energy of 12 nJ. Because of high SNR and Deoxy‐Hb‐dominant absorption, only the veins are visible. E) SLD‐PAM of the mouse ear sO_2_ using spectral unmixing. Wavelength: 532 and 558 nm. F) Comparison of the dual‐wavelength sO_2_ image and single‐wavelength Deoxy‐Hb image. G) LD‐PAM (Left) and SLD‐PAM (Right) of the mouse brain showing Deoxy‐Hb concentration. Wavelength: 675 nm. Laser pulse energy: 94 nJ. The brain image is acquired with the cranium intact. H) Schematic of the hypoxia challenge. J) Deoxy‐Hb‐PAM of mouse ear under three cycles of normoxia, hypoxia, and then returned to normoxia.

Enabled by much‐improved sensitivity, we develop a new approach for direct imaging of Deoxy‐Hb using a single‐wavelength (675 nm) low‐power pulsed laser. Compared with 532‐nm green light, the 675‐nm red light is 15–154‐fold less absorptive and thus is unlikely to suffer from the absorption saturation effect. We use 12‐nJ (**Figure** **4**C,D) and 32‐nJ (Figure S13B, Supporting Information) for in vivo Deoxy‐HB imaging in the microvessel. LD‐PAM barely distinguishes major vessels, and SLD‐PAM can observe the Deoxy‐Hb‐dominant veins but not the arteries. We compare them with conventional total hemoglobin concentration imaging and sO_2_ imaging acquired with two high‐absorptive wavelengths (Figure 4E; Figure S13A, Supporting Information). Results show that SLD‐PAM can selectively image Deoxy‐Hb dominant microvessels without spectral unmixing (Figure 4F). SLD‐PAM also shows superior Deoxy‐Hb imaging of the brain through the intact skull (Figure 4G). Because of low absorption and relatively low laser power, LD‐PAM images show almost no blood vessels. With the spectral‐spatial filter, many microvessels become visible. The single‐wavelength approach for Deoxy‐Hb quantification avoids wavelength‐dependent errors and noise amplification in spectral unmixing.^[^ ^44^, ^45^, ^46^ ^]^ The red light is less scattered than the green light in biological tissue. The high sensitivity of SLD‐PAM can overcome the challenge of low absorption and low laser power in the red spectrum and thus offer a new tool to map the oxygen level.

We monitored the hemodynamic responses by manipulating the oxygen concentration in the inhalation gas. The hypoxia challenge cycle was repeated three times to guarantee repeatability (Figure 4H). The response progress was compared through direct imaging of Deoxy‐Hb using a single wavelength and the spectral unmixing method using conventional dual wavelengths. At normoxia with 21% oxygen content, only the Deoxy‐Hb‐dominant veins (Scan1 in Figure 4J) are observed using single‐wavelength imaging. After switching the inhalation gas to the mixture of 5% oxygen and 95% nitrogen and was maintained it for 2 min, we observed enhanced photoacoustic signals in the artery. The phenomenon is because of the increased concentration of Deoxy‐Hb in arterial vessels. These hemodynamic changes will return to normal after recovering 6 min under normoxia. For the sO_2_ images, we can differentiate between main arteries and veins (Figure S14, Supporting Information). However, the sO_2_ levels in the artery decreased after the hypoxia challenge. Similar to single‐wavelength imaging, the physiological level will return to normal after recovering from the challenge.

### 2.4 Molecular Photoacoustic Imaging at Low Concentration

Photoacoustic microscopy can image a wealth of exogenous molecular or nano‐agents for cancer biology, neuroscience, regenerative medicine, and many other applications. An ideal photoacoustic contrast agent should have low toxicity, low photobleaching rate, strong absorption, and high photothermal efficiency.^[^ ^47^ ^]^ However, very few can meet all these requirements. For example, some inorganic nano‐agents have strong absorption but suffer from long‐term biotoxicity.^[^ ^48^, ^49^, ^50^ ^]^ Many organic nano‐agents and small molecules may have prominent biocompatibility but poor photostability.^[^ ^51^, ^52^, ^53^, ^54^ ^]^

Without complicating the engineering of new agents, SLD‐PAM enables molecular imaging with low‐power laser and low‐concentration agents, which can significantly reduce potential photobleaching and phototoxicity and lower the metabolic burden in clearing these exogenous agents.

**Figure 5:**
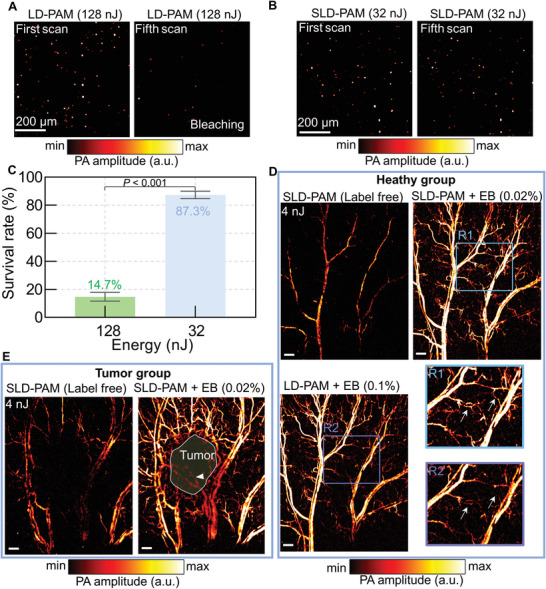
Molecular imaging at low laser energy and low concentration. A) Photoacoustic imaging of resorufin using 128‐nJ pulse energy. Bleaching is obvious after five times scanning. B) Photoacoustic imaging of resorufin using 32‐nJ pulse energy. Bleaching is much less than the 128‐nJ result. C) Calculated survival rates with high‐power and low‐power photoacoustic imaging. D) In vivo imaging of microvessels using 4‐nJ 620‐nm laser with the spectral‐spatial filter, enhanced with low‐concentration (with filter), and high‐concentration (without filter) Evans blue (EB). The arrows in the close‐up image indicate some representative improvements. SLD‐PAM uses five times fewer contrast agents and obtains more details. E) Photoacoustic imaging of tumor microvessels using 4‐nJ 620‐nm laser with the spectral‐spatial filter and enhanced by low‐concentration EB (with filter). The tumor boundary is better outlined with the low‐power laser and spectral‐spatial filter. Scale bars in D) and E) are 400 µm.

As a demonstration, we image Resorufin dye under two different laser pulse energies, 128 and 32 nJ, at 532 nm (see Experimental Section). Resorufin is a commercial fluorescent agent and has strong absorption in the visible spectrum. In conventional imaging with 128‐nJ pulse energy, after five times scanning, we observe strong photobleaching (**Figure** **5**A). The dye survival rate is only 14.7% (Figure 5C). Enabled by high sensitivity, SLD‐PAM can achieve nearly the same imaging quality with four times lower pulse energy (Figure 5B). The low laser power dramatically reduces photobleaching. After five times scanning with the low pulse energy, the dye survival rate increased to 87.3% (Figure 5C), ≈594% higher than the high‐energy one.

Evans blue (EB) is widely used in clinical and preclinical applications, such as total blood volume measurement, lymph node localization, capillary perfusion, and tumor imaging. We demonstrate that SLD‐PAM can significantly reduce the EB concentration in microvascular and tumor imaging. First, we imaged EB‐enhanced microvessels in the healthy mouse ear using 4‐nJ pulse energy at 620 nm. Without EB labeling, because hemoglobin has low absorption at 620 nm and the laser pulse energy is very low, we cannot see any blood vessels without using the spectral‐spatial filter. Using the spectral‐spatial filter, we can partially recover the trunk veins but not the arterials and the capillaries (Figure 5D). Because EB has a strong absorption at 620 nm, via injecting EB into the tail vein, we can enhance the vascular contrast. Without using the spectral‐spatial filter, we need to inject 0.1% (v/v) EB to boost the vessel signal. Enabled by the spectral‐spatial filter in SLD‐PAM, we can reduce the EB concentration to 0.02% (v/v) and acquire similar or even better images. Representative improved details, especially in branch vessels, are highlighted with arrows (Figure 5D). Without compromising the imaging quality, SLD‐PAM significantly lowers both laser pulse energy and dye concentration. Second, we imaged the subcutaneous tumor using a low‐power laser and reduced EB concentration. Blood vessels in and around the tumor are recovered, from which we can delineate the tumor boundary (Figure 5E).

## 3 Discussion

We present SLD‐PAM that offers up to 33 times higher sensitivity than the state‐of‐the‐art. We collaboratively improve the photoacoustic probe and innovate a 4D spectral‐spatial filter. Filtering coherent photoacoustic signals in all 3D space and all‐optical wavelengths significantly enhances weak signals. SLD‐PAM enables anatomical, functional, and molecular imaging with unprecedented super‐low laser pulse energy. Taking advantage of the much‐reduced laser pulse energy, broad‐spectrum imaging from green to red light is implemented based on a low‐cost low‐power broad‐spectrum laser. Various new anatomical, functional, and molecular imaging have been demonstrated in vivo.

With single‐digit nanojoules pulse energy, SLD‐PAM can quantify morphological parameters (diameter, tortuosity, and direction) and function parameters (sO_2_) in microvessels, which can minimize possible phototoxicity or perturbation to living tissues. We demonstrate its great potential in low‐power imaging of the brain and the eye. Utilizing the SLD‐PAM, it is feasible to adopt the low‐cost and lightweight low‐power light source. Therefore, developing wearable or portable OR‐PAM will become possible. In addition, compared with traditional OR‐PAM, SLD‐PAM will not be restricted by ANSI limits in high‐speed imaging environments (up to MHz). Long‐term monitoring of low‐absorbing or low‐concentration chromophores dynamically will become easier.

Enabled by much‐improved sensitivity, SLD‐PAM, for the first time, implements direct imaging of Deoxy‐Hb concentration using a low‐power red laser (675 nm). Direct imaging of Deoxy‐Hb concentration does not use spectral unmixing and thus avoids wavelength‐dependent errors and computation‐related noises. The single‐wavelength functional imaging also reduces system complexity and hardware cost and thus is more suitable for high‐speed functional imaging.

For molecular imaging, we show that the much‐reduced laser energy minimizes undesirable photobleaching by ∼85%. SLD‐PAM also allows for the use of lower‐concentration contrast agents, circumventing potential biotoxicity and metabolic burden for clearing. In addition, using regular laser energy or normal molecular concentration, SLD‐PAM can trade the high sensitivity for a broader selection of weak‐absorbing contrast agents.

Therefore, SLD‐PAM offers a new tool for low‐power anatomical, functional, and molecular imaging in preclinical studies and paves a new avenue for clinical translation.

## 4 Experimental Section

### Super‐Low‐Dose Photoacoustic Microscopy System

The SLD‐PAM system consisted of a low‐cost multi‐wavelength pulsed laser, a photoacoustic probe, a raster scanner, and a data acquisition and computation module (Figure 1A). The laser consisted of a nanosecond 532‐nm pump laser (VPFL‐G‐20, Spectra‐Physics) and a fiber‐based stimulated Raman scattering shifter. The pump laser operated at 4 or 8 kHz with a 7‐ns pulse width. Via tunning the pump laser pulse energy, the SRS shifter could provide at least 11 wavelengths from 532 to 695 nm (532, 545, 558, 570, 588, 604, 620, 640, 658, 675, and 695 nm). Three different laser systems for imaging endogenous and exogenous contrasts were demonstrated. They were a 532/558‐nm dual‐wavelength laser for vessel structure and oxygen saturation imaging (Figure S1A, Supporting Information), a 620‐nm laser for Evans blue (EB) imaging (Figure S1B, Supporting Information), and a 675‐nm laser for deoxyhemoglobin (Deoxy‐Hb) imaging (Figure S1C, Supporting Information). The detailed configuration description can be seen in Text S1 (Supporting Information). To improve the stability of the SRS laser, a shielding box was used to prevent the airflow into the optical system from outside as well as control the temperature fluctuation. The pulse energy fluctuations were not exceeding 20%. The pulsed light was delivered to the photoacoustic probe via single‐mode fiber. In the probe, a pair of achromatic doublets (AC064‐013‐A, Thorlabs, Inc.) focused the light onto the target. The acoustic waves were collected by an acoustic lens and an optical‐ultrasound beam combiner and detected by a high‐frequency ultrasound transducer (V214‐BC‐RM, Olympus‐NDT). The received signals were then amplified (48 dB, two ZFL‐500LN+ amplifiers, Mini‐Circuits) and digitized by a high‐speed data acquisition card at 500 MHz (ATS9360, Alazar Technologies) with 12‐bit resolution. The probe was raster scanned by two linear stages (PLS‐85, Physik Instrument).

### High‐Sensitivity Probe

To achieve high detection sensitivity, several new designs were made in the T‐probe. First, the acoustic lens with a large aperture (NA: 0.7160, Fused Silica) was fabricated to increase the acoustic collection efficiency. A detailed comparison of the HS‐probe (Figures S2A and S2B, Supporting Information) and T‐probe (Figures S2C and S2D, Supporting Information) was shown in both physical design and 2D numerical simulation.^[^ ^55^ ^]^ Figure S2E lists the acoustic materials properties of the HS‐probe. The noise equivalent pressure for the new HS‐probe was ≈0.097 mPa$\sqrt{Hz}$ ^−1^.^[^ ^56^ ^]^ Second, the gluing procedure was optimized to increase the acoustic transmittance in the optical/acoustic beam combiner, i.e., between the ultrasound transducer, the first prism, the second prism, and the acoustic lens. The key for gluing these parts was to cure the adhesive (NOA61 glue, Norland Products Inc) with ultraviolet and heating for over 24 h. The heating temperature was ≈70 °C. This procedure ensured firm bonding and low stress in the assembled optical/acoustic beam combiner, resulting in increased acoustic transmittance. Third, to precisely align the acoustic and optical foci, the optical/acoustic parts and assembly were redesigned to minimize the deformation‐caused misalignment. The incident optical path was vertical so that gravity causes minimal deformation of the optical beam and reduces misalignment between the optical and acoustic foci. In the previous design, the acoustic/laser focal zone usually occurred a deviation induced by the gravity effect after experiencing several times scanning.

### Spectral‐Spatial Filter Algorithm

A‐line signals are time‐variant and map the absorber distributions along the depth direction. Because the photoacoustic signals have consistency and continuity within a 3D sub‐volume (or called block), correlated 3D blocks could be matched in the spatial domain. Moreover, volumetric images at different optical wavelengths were usually highly correlated, thus spatially registered 3D blocks at different optical wavelengths could also be matched. These spectral‐spatial block‐matched data were collected and a collaborative filter was used to suppress the noises to the best extent. The algorithm is schematically depicted in Figure 1C. Normalization was first applied to the volumetric datasets at each optical wavelength. The denoising process consisted of three steps: grouping, collaborative filtering, and re‐assigning. In the first step of grouping, a reference cubic sub‐volume at one optical wavelength was designated. The side length of the cube was set to 10 µm, close to the capillary diameter. Sub‐volumes similar to the reference block were selected in the neighboring region according to a photometric distance criterion. If the photoacoustic datasets were acquired at more than one wavelength, sub‐volumes at other optical wavelengths were selected from their volumetric datasets using the same spatial coordinates. A set of matched 3D blocks from all optical wavelengths were stacked together for grouping. In the second step of filtering, four linear transformations were applied to the grouped blocks. Three of them were biorthogonal spline wavelet (bior1.5) transformations on the three spatial dimensions. The remaining one was a 1‐D Haar wavelet transformation on the fourth dimension.^[^ ^39^ ^]^ Threshold truncation was applied in the transformation domain to eliminate electrical, thermal, or any other noises. The filtered data set was restored to the spectral‐spatial domain via four corresponding inverse linear transformations. In the third step of re‐assigning, the filtered sub‐volumes were assigned back to their original spectral‐spatial coordinates.

The three steps were repeated on different reference sub‐volumes that were sequentially traversed with a step size of 5 µm over the volumetric dataset. One problem was that repeated filtering may generate multiple sub‐volume data at one position and cause overlapping. To address this problem, weighted averaging was used to combine the overlapped sub‐volume data. The weights were determined by the standard deviation of the volumetric data and the number of non‐zero coefficients of the corresponding block set. The detailed mathematics is described in Text S2 (Supporting Information). All data were processed in MATLAB (R2019b, MathWorks, USA) on a computer (Inter Core i7@2.60 GHz, 16 GB of RAM, NVIDIA GeForce RTX 2060).

### Sensitivity Characterization

The sensitivity of T‐PAM and LD‐PAM was compared. The two systems were used to image a black tape sample with the same laser energy. In vivo vasculature 2D/3D imaging (mouse ear) was further conducted to evaluate the sensitivity improvement from T‐PAM to LD‐PAM (Figure S5, Supporting Information). Because the sensitivity improvement from LD‐PAM to SLD‐PAM may vary on different pixels, the in vivo imaging quality was directly compared at different laser pulse energies (mouse ear and brain). The structural similarity index measure (SSIM)^[^ ^57^ ^]^ was used as a global parameter to evaluate the average sensitivity improvement of SLD‐PAM (Figure S7, Supporting Information) and the signal‐to‐background ratio (SBR) was used to determine the local region improvement (Figure S8, Supporting Information). The SBR was defined as the ratio of the maximal signal amplitude and the mean background amplitude. In the SBR calculation, the images for the division were acquired with the same laser pulse energy. In the SSIM calculation, the reference image (Figure S6, Supporting Information) was acquired with the HS‐probe at high laser pulse energy but not processed with the spectral‐spatial filter. The SSIM ratio was calculated within eight nanojoules, and the laser pulse energy in LD‐PAM was four times as much as in SLD‐PAM.

### Super‐Low‐Dose Imaging of the Microvessels and Oxygen Saturation

In the vascular morphology imaging, photoacoustic images from the ear, the eye, and the brain were acquired using the 532‐nm laser at different pulse energies from 0.5 nJ to 64 nJ. In the mouse ear, two wavelengths (532 nm and 558 nm) were also used for sO_2_ measurement at these pulse energies. The nanosecond pulses at the two wavelengths were switched with a 300‐ns delay to avoid signal overlapping. Dominant absorbers at the two wavelengths were Oxy‐Hb and Deoxy‐Hb. In spectral unmixing, the dual‐wavelength volumetric data were first processed using the spectral‐spatial filter algorithm. Then, a linear model was used to unmix the spectrum. The detailed implementation can be found in Text S3 (Supporting Information). Enabled by the high sensitivity, a low‐power 675‐nm laser was used to image Deoxy‐Hb in the ear (12 nJ and 32 nJ) and the brain (94 nJ).

Vascular morphological parameters, such as tortuosity, direction, and diameter. First, the maximum amplitude projection (MAP) image of a denoised volumetric dataset was computed. A threshold, 5% of the maximal amplitude, was applied to the MAP image to remove small noises. Then a multi‐scale Hessian‐based Frangi vessel filter was applied to enhance the image contrast.^[^ ^58^, ^59^, ^60^, ^61^ ^]^ Next, local first‐order statistics were used to adaptively compute the threshold at every pixel to convert the MAP image into a binary image. Finally, a routine vasculature processing pipeline,^[^ ^62^ ^]^ including vascular segment skeletonization, spline‐fitting, and vessel edge identification, was implemented. The tortuosity was determined from the ratio of the actual vascular segment length and the linear distance between its two ends. The direction was defined as the angle between the vascular segment and the positive horizontal axis. The diameter was determined from the shortest distances between the centerline and both sides of the vessel segment.

### Exogenous Molecular Preparation

For many molecular or nano‐agents, high laser energy or high molecular concentration may cause bleaching or biotoxicity problems. Enabled by SLD‐PAM, in vitro and in vivo molecular imaging was demonstrated with much‐reduced bleaching and molecular concentration. For in vitro imaging, resorufin (C_12_H_6_NNaO_3_) was imaged with different laser powers to show reduced photobleaching.^[^ ^63^ ^]^ Resorufin was dissolved in phosphate‐buffered saline (PBS) and Matrigel (1:1) to produce a 100‐µm sample. The sample was uniformly smeared on coverglass. A control group was a 1:1 mixture of PBS and Matrigel. The laser wavelength was 532 nm, and the laser pulse energy was 128 and 32 nJ for high‐dose and low‐dose imaging. The samples were repetitively imaged with the PAM system multiple times. Photoacoustic amplitudes from three samples were analyzed to calculate the survival rates of the molecules. The survivors were defined as the total pixel photoacoustic amplitude exceeding a preset threshold.

For in vivo imaging, low‐concentration EB dye was injected into healthy and tumor‐bearing mice via the tail vein.^[^ ^64^ ^]^ A 620‐nm pulsed laser was used to image EB. EB solution (0.5%, w/v; Phygene, Fuzhou, Fujian, China) was prepared and different volumes (75 µL Versus 375 µL) were injected into different mice. The body weight of a mouse was ≈25 g. The concentration of EB in the bloodstream was ≈0.02% (v/v) and 0.1% (v/v), respectively.

### Animal Preparation

All animal procedures were approved by the animal ethical committee of the City University of Hong Kong (20‐201 in DH/HT&A/8/2/5 Pt.3). Female ICR mice (≈25 g, 3–4 weeks old) were used for ear, eye, and brain imaging. The imaging qualities of T‐PAM, LD‐PAM, and SLD‐PAM were compared. In imaging, mice were anesthetized via breathing in 1.5% (v/v) vaporized isoflurane. For ear imaging, hairs were gently removed with a depilatory. For brain imaging, the scalp was removed with the cranium intact. The mice were immobilized using a stereotactic head holder. Ultrasound gel was applied on the tissue surface for acoustic coupling. A water tank containing deionized water was placed on top of the tissue and ultrasound gel.

A tumor‐bearing mouse model (BALB/c, 3–4 weeks old) was used in the low‐concentration molecular imaging. 4T1 breast cancer cells (1 million cells in 20‐µL PBS) were injected into the mouse ear. In 7 days, a ≈1.7‐mm^3^ tumor grew in the mouse ear.

A three‐cycle hypoxia challenge was conducted. In the first cycle, the mouse was anesthetized with 1.5%, v/v, isoflurane, and vessels on the ears were imaged. The oxygen content in inspiratory air was ≈21%. After completing the scanning, the inspiratory air was changed to 5% oxygen and 95% nitrogen and maintained for 2 min. The resultant physiological changes induced by hypoxia were recorded by the SLD‐PAM and inspiratory oxygen content was recovered to the initial level until the scanning is over. The next cycle started after a total of 6 min recovery.

For molecules imaging, healthy and tumor‐bearing mice were imaged using a 620‐nm wavelength with 4‐nJ pulse energy. In the healthy group, first, 75‐µL EB solution (0.5% w/v) was injected via the tail vein. Data were immediately acquired after injection. Then the injection doses were increased to 375 µL for comparison. In the tumor‐bearing group, 75‐µL EB solution was injected via the tail vein to enhance the visibility of the blood vessels.

The fluence used in all animal experiments was much lower than the MPE. The MPEs for single‐wavelength and dual‐wavelength OR‐PAM on the skin are presented in Text S5 (Supporting Information).

### Statistical Analysis

The acoustic simulation to compare HS‐probe and T‐probe was carried out using the k‐Wave toolbox. Before implementing the spectral‐spatial filter, normalization was first applied to the volumetric datasets at each optical wavelength. The experimental data in Figure 5C were presented as mean ± standard deviation. Three different samples were used for the analysis. Statistic significances for two‐group comparisons were determined by two‐tailed Student's *t*‐tests. Statistical tests were done using GraphPad Prism (v8.0.1a) (GraphPad, USA). Image reconstruction, processing, and analysis were performed on MATLAB (R2019b) (MathWorks, USA).

## Conflict of Interest

The authors declare no conflict of interest.

## Supporting information

Supporting Information

Supplemental Movie 1

## Data Availability

The data that support the findings of this study are available from the corresponding author upon reasonable request.
