# Supplementary material for: Super‐Low‐Dose Functional and Molecular Photoacoustic Microscopy
Source: Adv Sci (Weinh). 2023 Jun 13;10(23):2302486. doi: 10.1002/advs.202302486 (PMC10427362; doi:10.1002/advs.202302486)
Supplement: Supplementary file 1 — Supporting Information [file ADVS-10-2302486-s001.pdf]

ADVANCED SCIENCE
Open Access

Supporting Information

for *Adv. Sci.*, DOI 10.1002/advs.202302486

Super-Low-Dose Functional and Molecular Photoacoustic Microscopy

*Yachao Zhang, Jiangbo Chen, Jie Zhang, Jingyi Zhu, Chao Liu, Hongyan Sun and Lidai Wang**

Supporting Information

Super-low-dose functional and molecular photoacoustic microscopy

Yachao Zhang[1#], Jiangbo Chen[1#], Jie Zhang[2], Jingyi Zhu[1], Chao Liu[1], Hongyan Sun[2], Lidai Wang[1,3*]

*Corresponding author. Email: lidawang@cityu.edu.hk

This PDF file includes:

Figures S1 to S14
Legend for movie S1
Supplementary Text

Other Supplementary Information for this manuscript include the following:

Movie S1

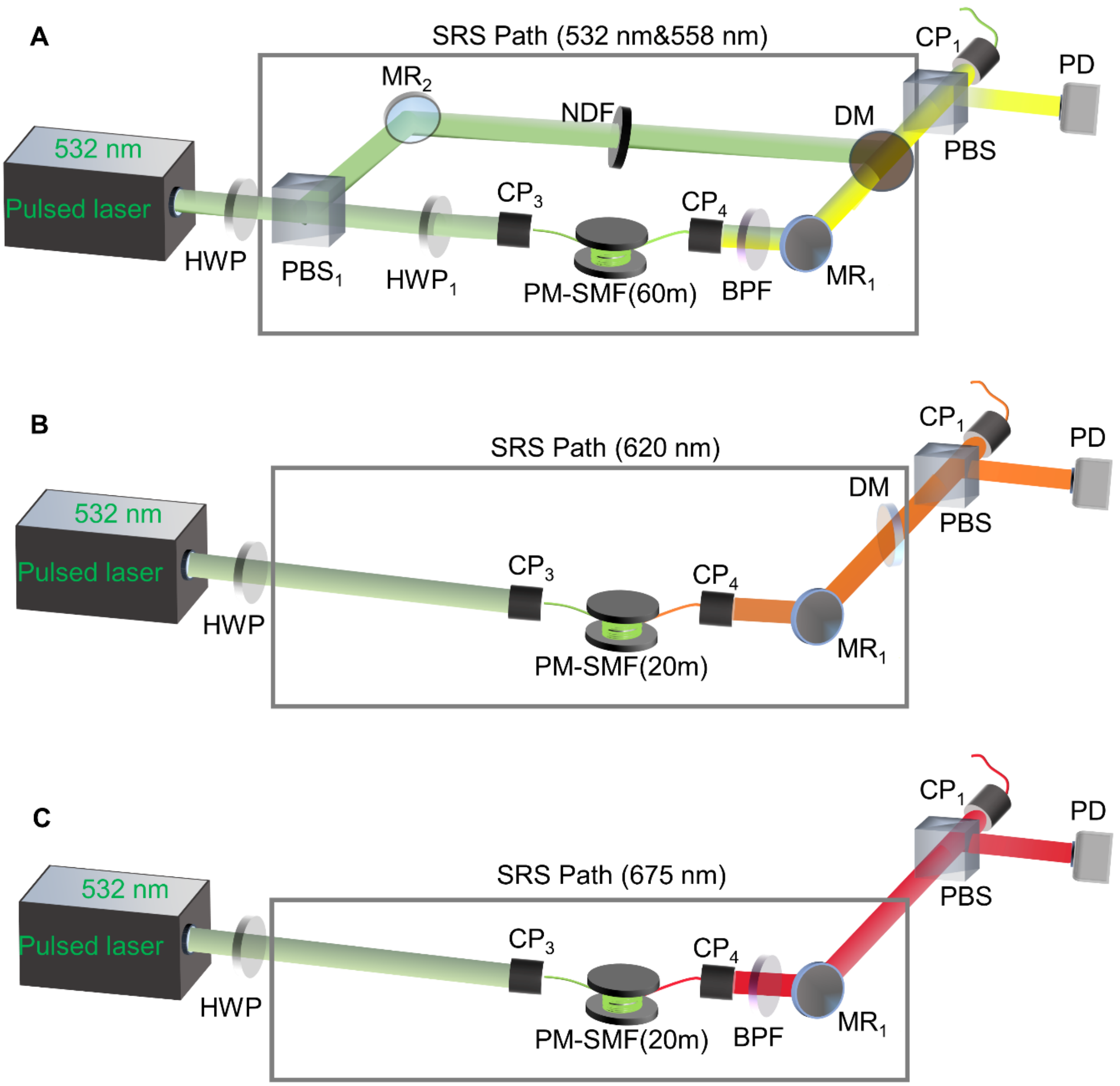

Figure S1. Representative multi-wavelength lasers used in the SLD-PAM system. A) Dual-wavelength of 532 nm and 558 nm laser source for vascular morphology and oxygen saturation (sO_2) imaging. B) Single wavelength of 620 nm laser source for the exogenous molecular imaging (Evans blue). C) Single wavelength of 675 nm laser source for the deoxyhemoglobin (Deoxy-Hb) imaging. BPF, bandpass filter; CP, coupler; DM, dichroic mirror; HWP, half-wave plate; MR, mirror; NDF, neutral density filter; PBS, polarizing beam splitter; PD, photodiode; PM-SMF, polarization-maintaining single-mode fiber; SRS, stimulated Raman scattering.

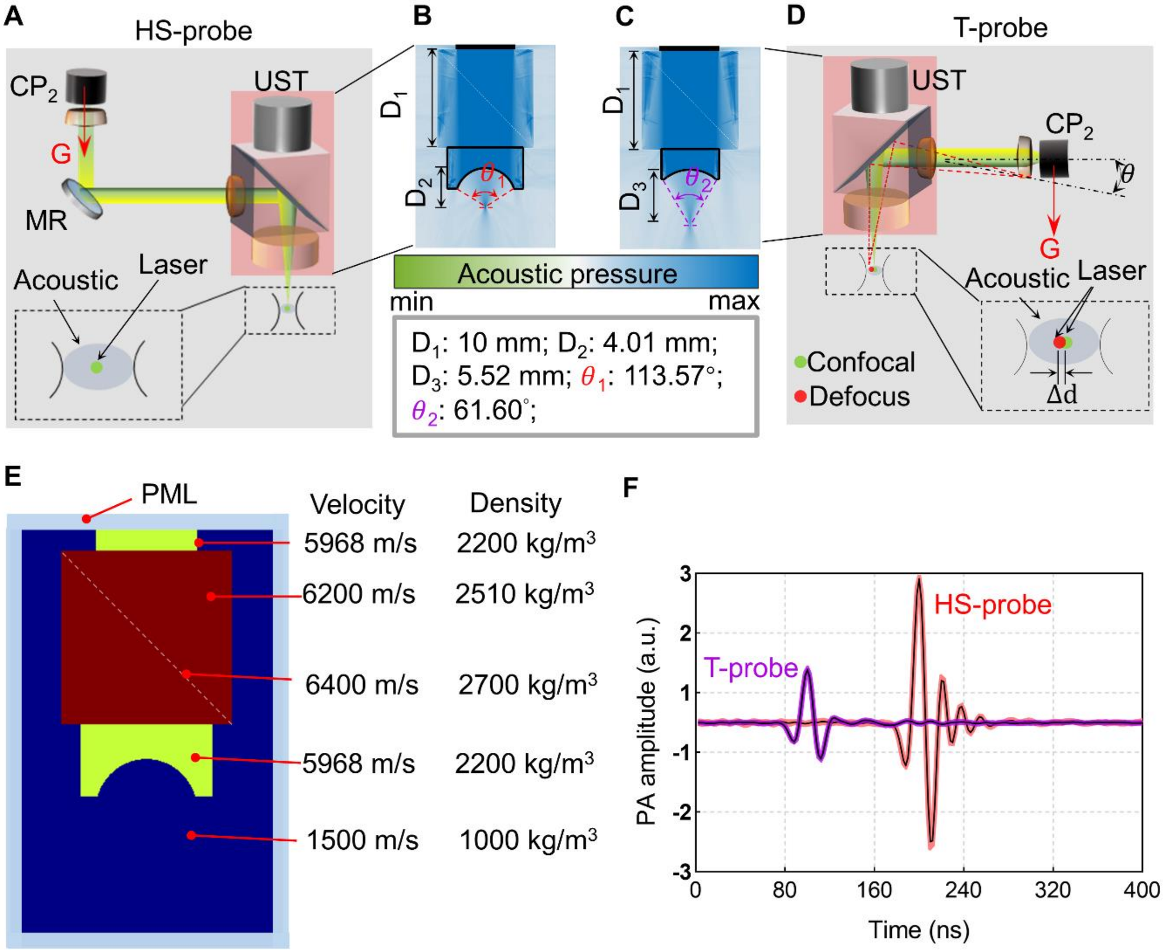

Figure S2. Comparison of the HS-probe and T-probe. A) Schematic of the HS-probe. The maximal acoustic pressure field simulated by the B) HS-probe and C) T-probe. D) Schematic of the T-probe. E) Acoustic materials properties of the HS-probe. F) Experimental sensitivity comparison between T-probe (T-PAM) and HS-probe (LD-PAM). CP, coupler; G, gravity; MR, mirror; UST, ultrasound transducer.

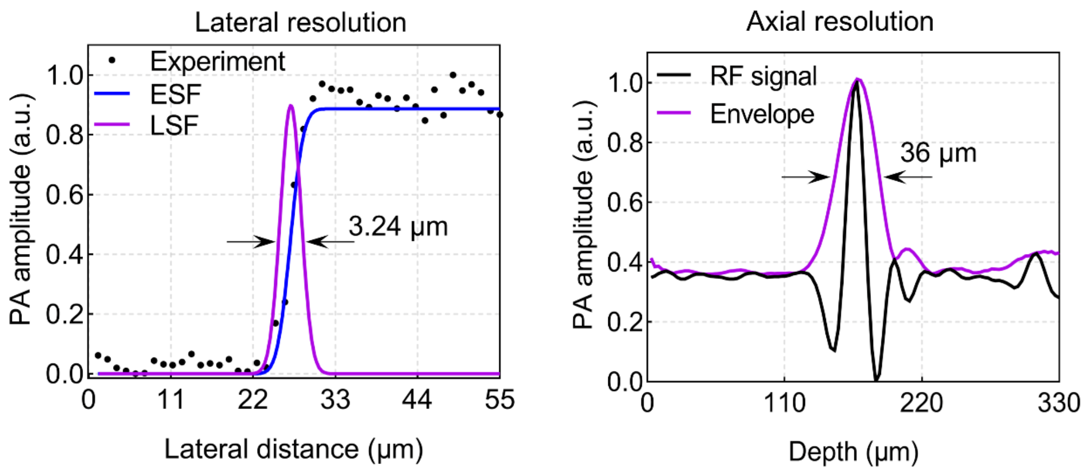

Figure S3. Spatial resolution of the SLD-PAM.

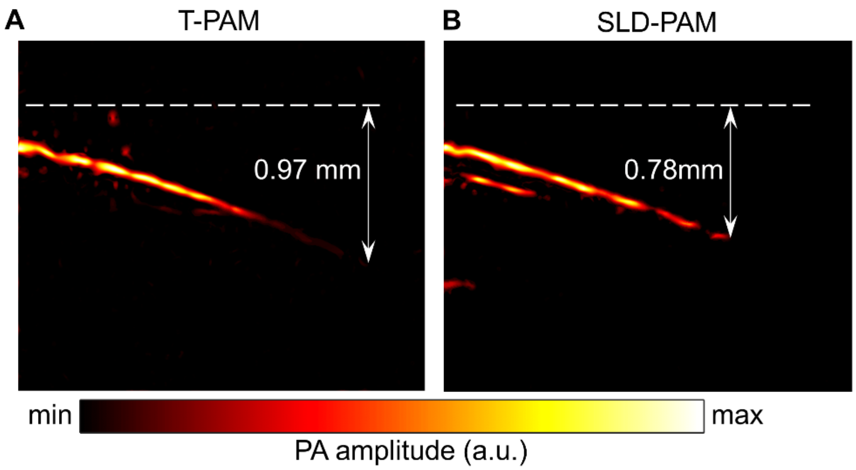

Figure S4. Maximal imaging depth of the T-PAM and SLD-PAM.

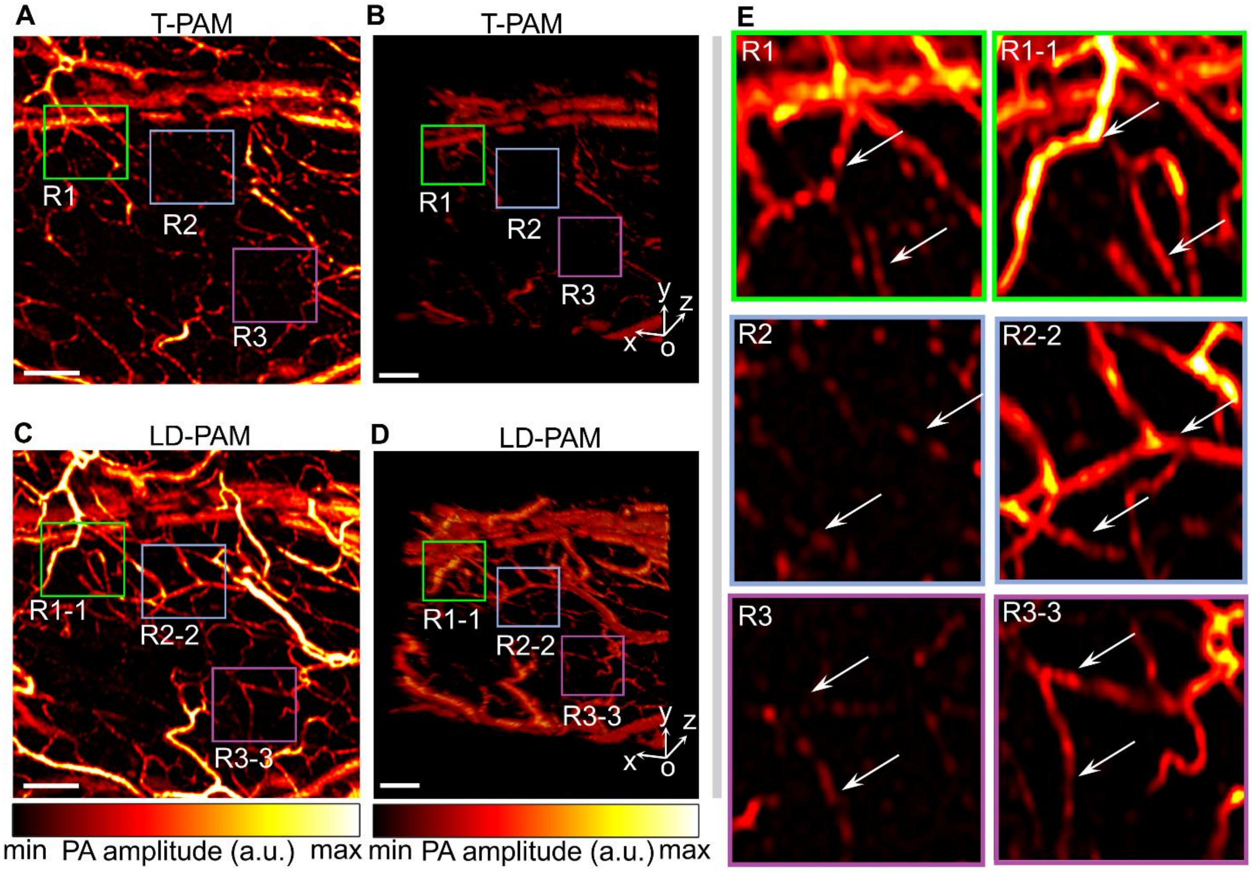

Figure S5. Comparison of vasculature imaging acquired by T-PAM and LD-PAM. A) Vasculature image acquired by T-PAM and B) its corresponding 3D result. C) Vasculature image acquired by LD-PAM and D) its corresponding 3D result. E) The close-up of regions marked in A) and C). The arrows show the improved details and signal-to-noise ratio. The wavelength used was 532 nm and the scale bars are 200 μm.

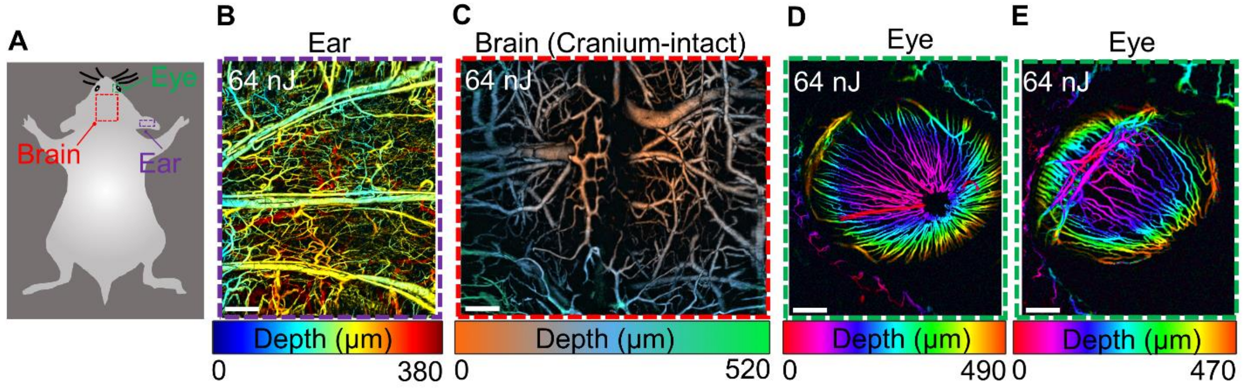

Figure S6. Depth-encoded photoacoustic images acquired by LD-PAM. A) Illustration of the regions of interest. Depth-encoded photoacoustic images of B) mouse ear, C) brain with cranium intact, and D-E) eyes from different scanning perspectives. The wavelength used was 532 nm and the scale bars are 400 μm.

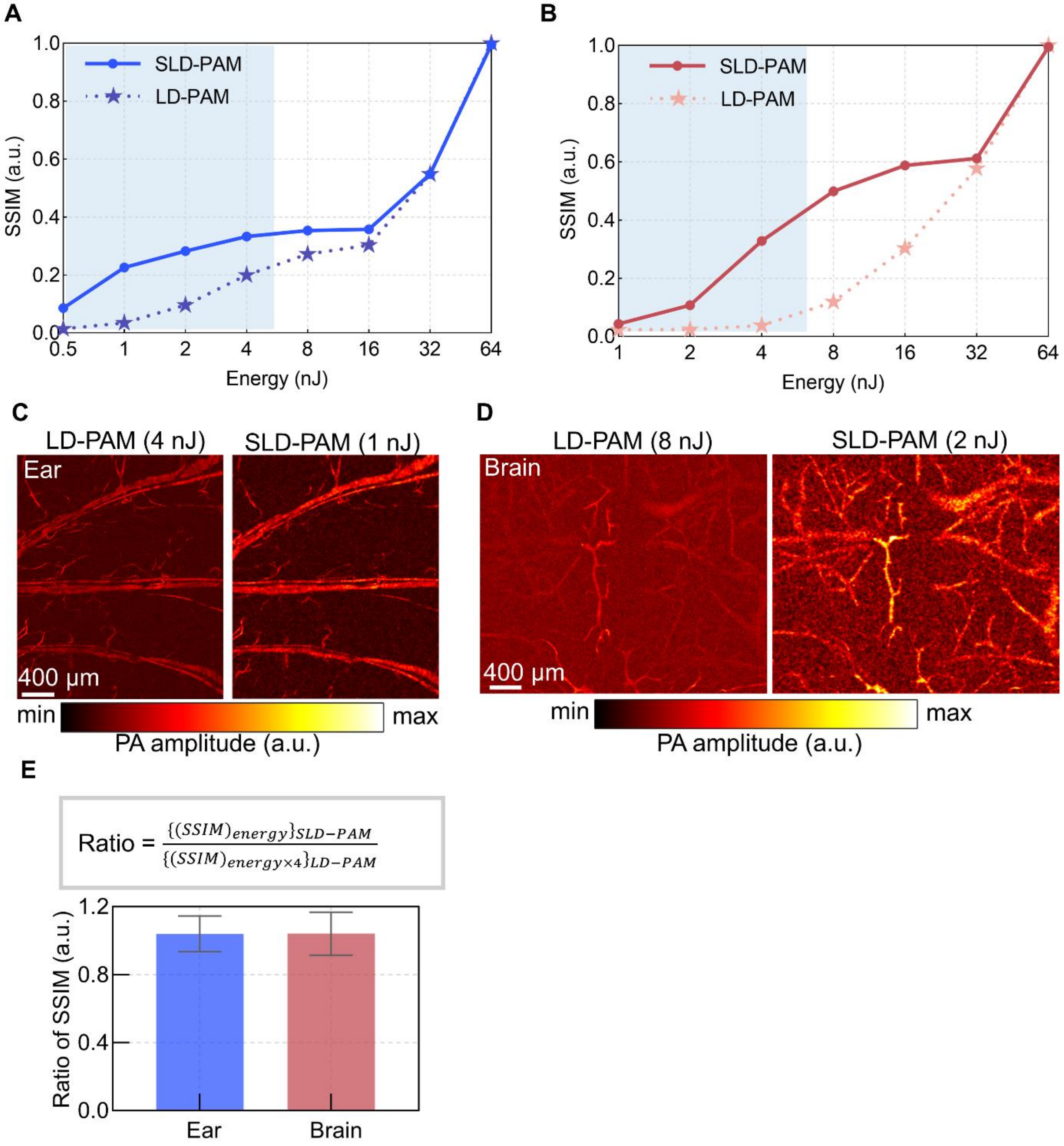

Figure S7. Comparison of average sensitivity between LD-PAM and SLD-PAM. The SSIM values as a function of laser energy were calculated from photoacoustic images of A) mouse ear and B) mouse brain. Comparison of photoacoustic images of C) mouse ear and D) mouse brain acquired by LD-PAM under high laser power and by SLD-PAM under low laser power. E) The SSIM ratio between SLD-PAM and LD-PAM within the energy range shadowed in A) and B). The laser doses in LD-PAM are 4 times as much as in SLD-PAM. The referenced image for all SSIM calculations is acquired by LD-PAM at a laser dose of 64 nJ (Figure S6). The wavelength used was 532 nm.

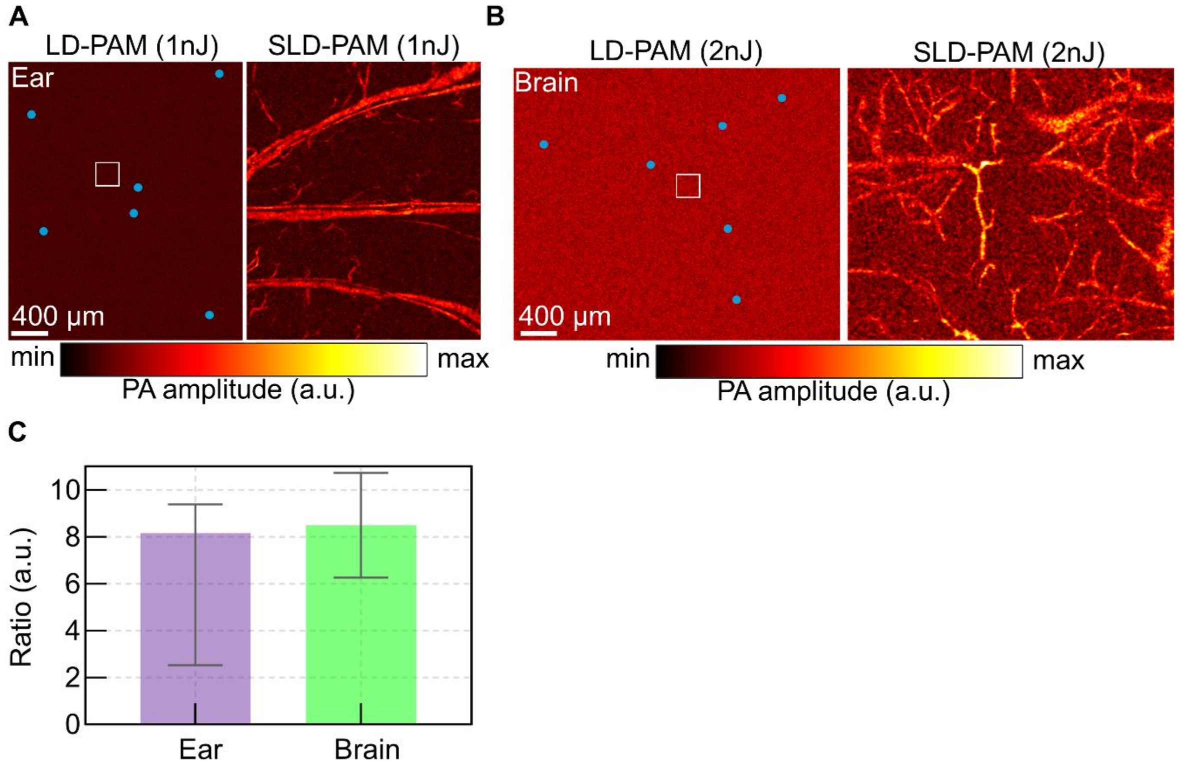

Figure S8. Comparison of the local sensitivity between LD-PAM and SLD-PAM. Comparison of photoacoustic images of A) mouse ear and B) mouse brain acquired by LD-PAM and SLD-PAM under the same laser dose. The blue solid circles represent regions of blood vessel signals, and the white rectangle shows the regions of background noise. C) The SBR ratio between SLD-PAM and LD-PAM. The signal background ratio (SBR) is defined as the ratio of maximal signal amplitude and mean background noise. The SBR ratio is defined as $\{\frac{\max(I_{signal})}{mean(I_{bg})}\}_{SLD-PAM} \times \{\frac{mean(I_{bg})}{\max(I_{signal})}\}_{LD-PAM}$). The wavelength used was 532 nm.

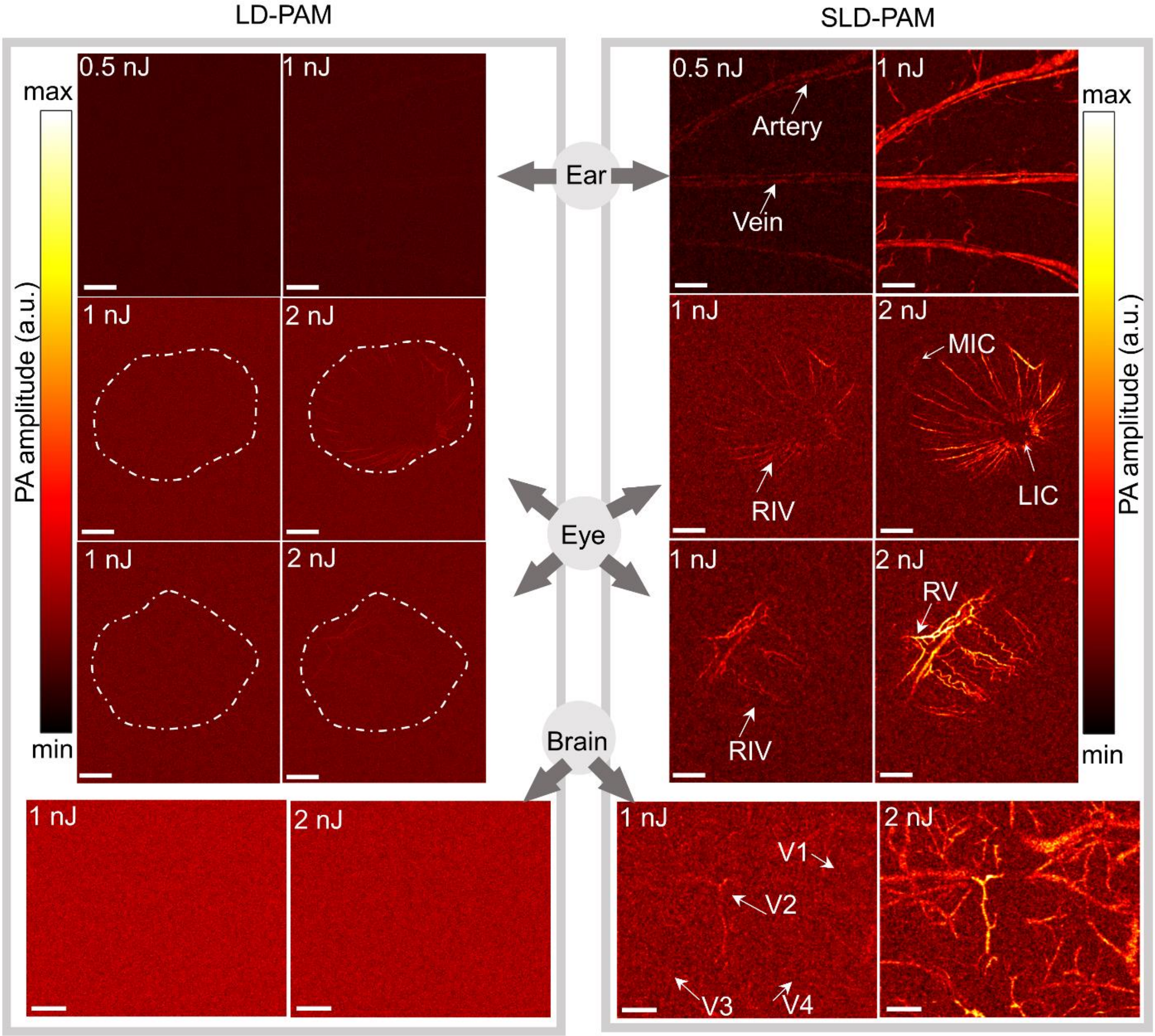

Figure S9. Comparison of photoacoustic images of the mouse ear, eye, and cranium-intact brain acquired by LD-PAM and SLD-PAM with super-low pulse energy. LIC, lesser iris circle; MIC, major iris circle; RV, retinal vessel; RIV, radial iris vessel; The arrows show the improved details. The wavelength used was 532 nm and the scale bars are 400 μm.

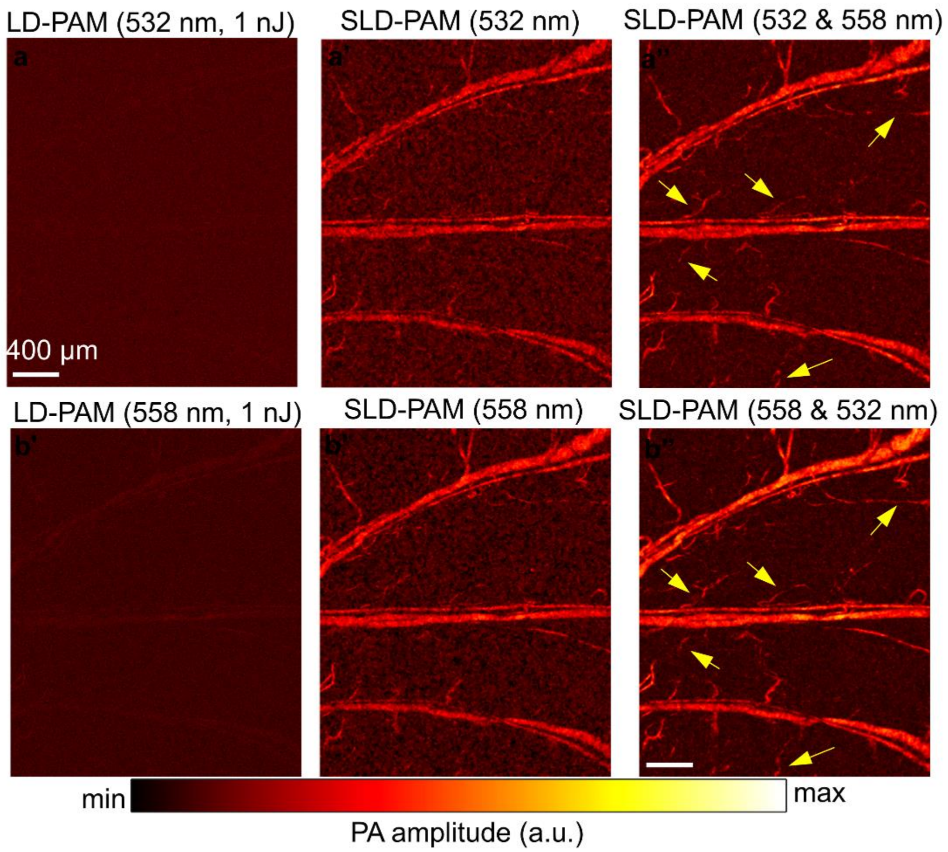

Figure S10. A full comparison of raw images and filtered results at different wavelengths.

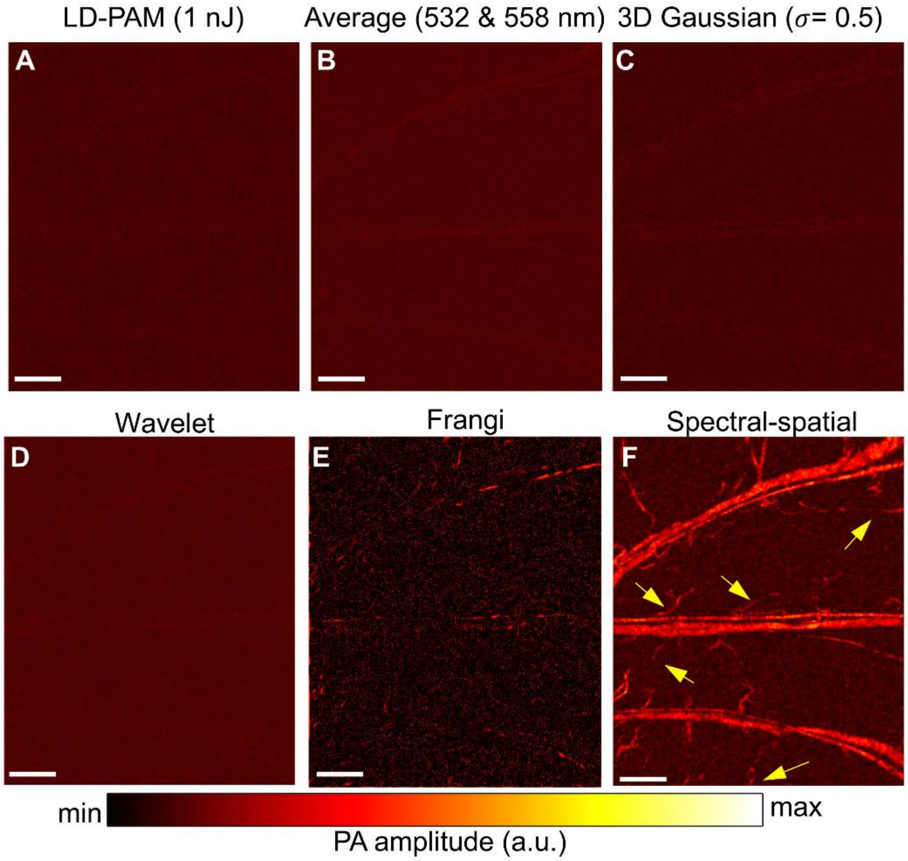

Figure S11. Comparison of the spectral-spatial filter with other denoised algorithms. A) vasculature imaging acquired by LD-PAM. The results processed by the B) dual-wavelength average (532 nm and 558 nm), C) 3D Gaussian filter, D) Wavelet filter, E) Frangi filter, and F) spectral-spatial filter. The arrows show the improved details. The wavelengths used for spectral-spatial filter were 532 nm and 558 nm. The laser energy used is 1 nJ. The scale bars are 400 μm.

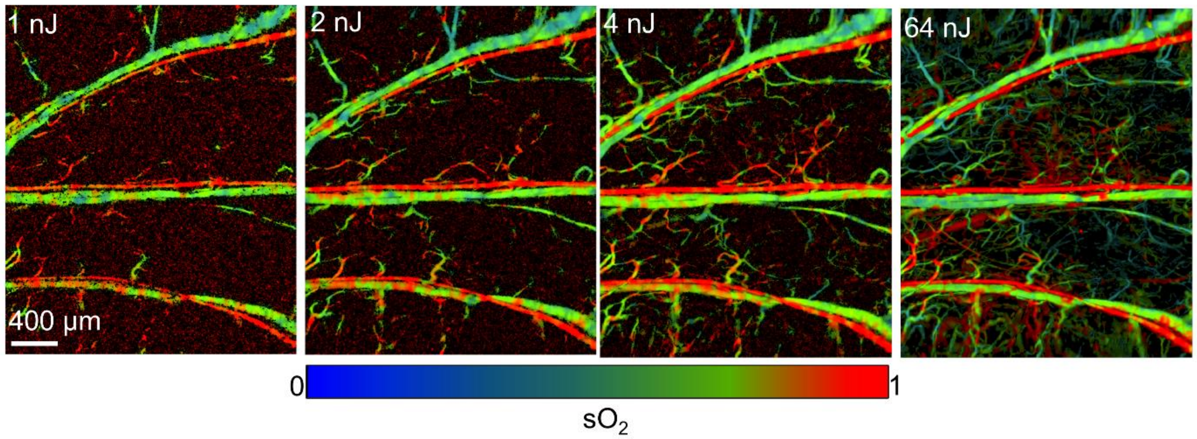

Figure S12. Comparison of the oxygen saturation (sO_2) acquired by SLD-PAM under different laser pulse energies**.** The wavelengths for spectrum unmixing were 532 nm and 558 nm.

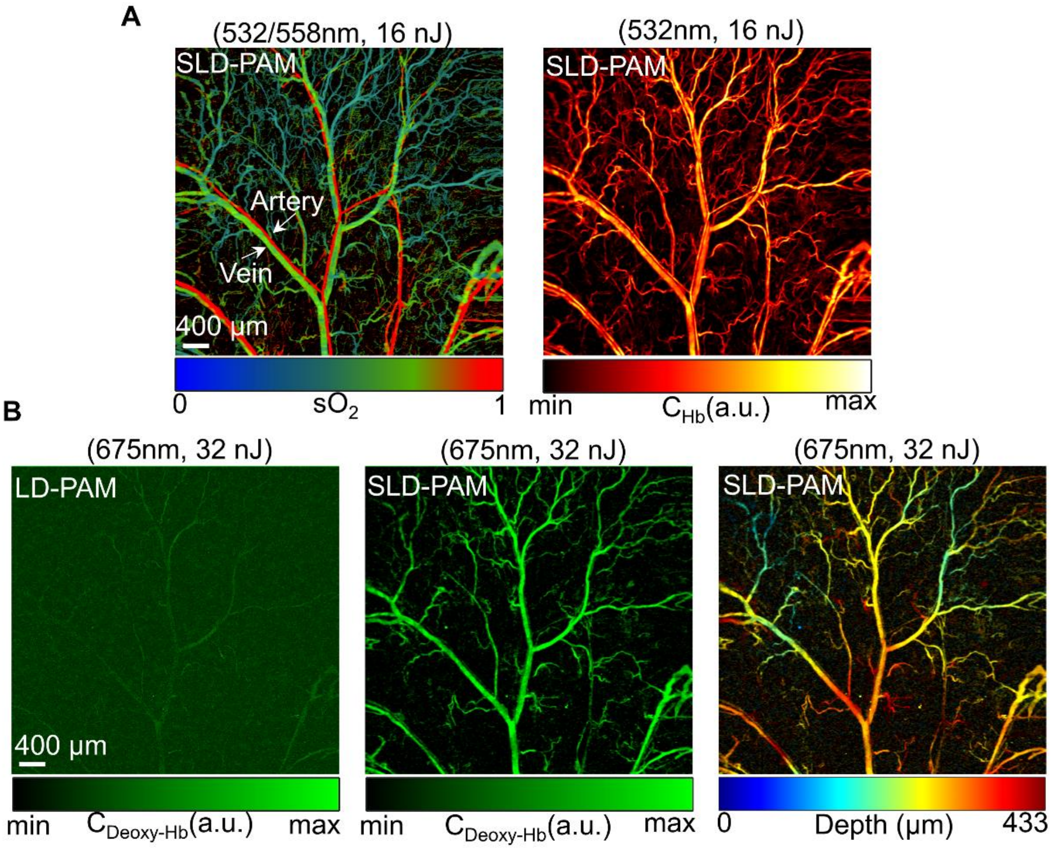

Figure S13. Comparison of the photoacoustic images of oxygen saturation (sO_2), total hemoglobin (HbT) concentration, and deoxyhemoglobin (Deoxy-Hb) concentration using the SLD-PAM. A) SLD-PAM imaging of the mouse ear sO_2 using spectral unmixing and the HbT concentration using single wavelength. Wavelength: 532 and 558 nm. B) SLD-PAM imaging of the mouse ear showing Deoxy-Hb concentration only. Left: raw image; Middle: with spectral-spatial filter; Right: depth-encoded vasculature image. Wavelength: 675 nm. Pulse energy: 32 nJ.

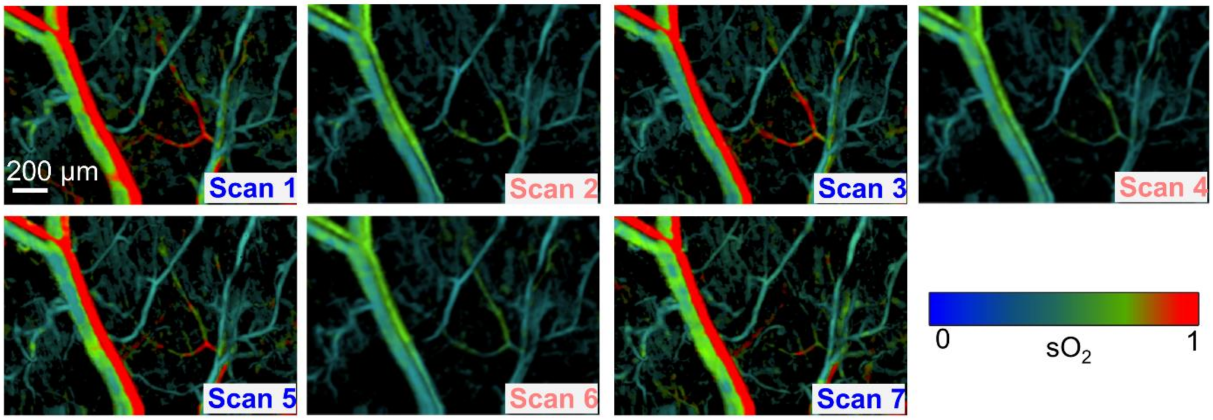

Figure S14. SLD-PAM of the mouse ear sO_2 under three cycles of normoxia, hypoxia, and then returned to normoxia.

Movie S1. Comparison of vascular imaging at 1-nJ laser pulse energy acquired by LD-PAM and SLD-PAM. The tracked green solid circle records the corresponding A-line signal at different positions.

Supplementary Text

Supplementary Text S1. Multi-wavelength low-cost laser systems

For the dual-wavelength optical path, the output laser beam from a pump laser (VPFL-G-20, Spectra-Physics) is split into two paths by a polarization beam splitter (PBS, PBS051, Thorlabs, Inc.), and the energy ratio of the two paths is adjusted with a half-wave plate (HWP, GCL-060633, Daheng Optics). One path transmits in free space, and the other one is coupled into a 60-m polarization-maintaining single-mode fiber (PM-SMF, HB450-SC, Fibercore Limited). Via stimulated Raman scattering (SRS), the 532-nm pulse in the 60-m fiber generates a 558-nm pulse with a 300-ns delay. An HWP is placed before the 60-m PM-SMF to adjust the polarization of the 532-nm light to maximize the SRS efficiency. The 532-nm beam is combined with the delayed 558-nm beam using a dichroic mirror (DM, T550lpxr-UF1, Chroma Technology), and the dual-wavelength is coupled into a 2-m PM-SMF (P1-460Y-FC, Thorlabs).

For the 620-nm and 675-nm lasers, the pump laser beam is coupled into a 20-m PM-SMF (HB450-SC, Fibercore Limited), and the HWP is placed before the 20-m PM-SMF to adjust the polarization of the pump laser to adjust the SRS efficiency. Two different optical filters are used to pass the 620 nm (#67-083 dichroic filter, Edmund Optics) and 675 nm (ET675/20m bandwidth filter, Chroma) wavelengths. The filtered laser beam is coupled into a 2-m PM-SMF for photoacoustic imaging.

Supplementary Text S2. Mathematics implementation of the spectral-spatial filter

V_{λ_1} and V_{λ_2} represent the volumetric data acquired at different optical wavelengths of λ_1 and λ_2, respectively. The normalized volumetric data become

$$\bar{V}_{\lambda_1} = V_{\lambda_1}/\max(V_{\lambda_1}, V_{\lambda_2}) \\ \bar{V}_{\lambda_2} = V_{\lambda_2}/\max(V_{\lambda_1}, V_{\lambda_2}) \tag{S-1}$$

Then the wavelength-dependent volumetric data are jointly processed. We firstly selected the referenced sub-volume $\langle S_{\lambda_1}\rangle_{x_R}$ from $\bar{V}_{\lambda_1}$. x_R is the spatial coordinate of the reference sub-volume $\langle S_{\lambda_1}\rangle_{x_R}$, which is used to determine a reference sub-volume $\langle S_{\lambda_2}\rangle_{x_R}$ from $\bar{V}_{\lambda_2}$. Sub-volumes $\langle S_{\lambda_1}\rangle_{x_i}$ and $\langle S_{\lambda_2}\rangle_{x_i}$ that are spatially close to and similar to the reference sub-volumes

$$\langle S_{\lambda_1}\rangle_{x_R}^{ht} = \{x_i \epsilon X_{\lambda_1}, \frac{\left\| \langle S_{\lambda_1}\rangle_{x_i} - \langle S_{\lambda_1}\rangle_{x_R} \right\|_2^2}{L\times L\times L}\} < \tau_{match}\} \\ \langle S_{\lambda_2}\rangle_{x_R}^{ht} = \{x_i \epsilon X_{\lambda_2}, \frac{\left\| \langle S_{\lambda_2}\rangle_{x_i} - \langle S_{\lambda_2}\rangle_{x_R} \right\|_2^2}{L\times L\times L}\} < \tau_{match}\} \tag{S-2}$$

where X confines the spatial domain of the volumetric data, i represents matched sub-volume index in a sub-volume set, L is the size of the reference sub-volume, and τ_{match} is the pre-defined threshold value to search similar sub-volumes of $\langle S_{\lambda_1}\rangle_{x_i}$ and $\langle S_{\lambda_2}\rangle_{x_i}$.

Then a 4D data is formed by matrix disjoint union of all similar sub-volumes at all optical wavelengths,

$$G_j^4 = \coprod_{x_i \epsilon \langle S_{\lambda_1}\rangle_{x_R}^{ht},\, or\ \langle S_{\lambda_2}\rangle_{x_R}^{ht}} \langle S_{\lambda_1}\rangle_{x_i} \langle S_{\lambda_2}\rangle_{x_i} = [\langle S_{\lambda_1}\rangle_{x_1}, \langle S_{\lambda_1}\rangle_{x_2}, \cdots, \langle S_{\lambda_1}\rangle_{x_i}, \cdots, \langle S_{\lambda_2}\rangle_{x_1}, \langle S_{\lambda_2}\rangle_{x_2}, \cdots, \langle S_{\lambda_2}\rangle_{x_i}] \tag{S-3}$$

where j represent a 4D data G_j^4, $\coprod$ is disjoint union operation (38).

A collaborative filter, including four separable linear transformation T_4 (Wavelet toolbox, MATLAB 2019b, MathWorks, USA), a coefficients shrinkage operator γ^{ht}, and four inverse transformation T_4^{-1}, are written as

$$\hat{G}_j^4 = \coprod \langle \bar{S}_{\lambda_1}\rangle_{x_i} \langle \bar{S}_{\lambda_2}\rangle_{x_i} = T_4^{-1}(\gamma^{ht}(T_4(G_j^4))) \tag{S-4}$$

where $\hat{G}_j^4$ represents filtered G_j^4, and $\langle \bar{S}_{\lambda_1}\rangle_{x_i}$ and $\langle \bar{S}_{\lambda_2}\rangle_{x_i}$ are the filtered sub-volumes. T_4 includes a 3D biorthogonal spline wavelet (bior1.5) on the cubic, and a 1-D Haar wavelet on the fourth dimension. The coefficients shrinkage is operated by threshold truncation.

Finally, $\hat{G}_j^4$ is re-assigned to their original positions of both volumetric data. Sub-volume data from different 4D groups or within the same group may be overlapped. Weighting factors $W_{x_R}^{\lambda_i}$ are used to generate new averaged volumetric data as

$$\bar{V}_{\lambda_1}^{ht} = \frac{\sum_{x_R}(\sum_{x_i} W_{x_R}^{\lambda_1} \langle \bar{S}_{\lambda_1}\rangle_{x_i})}{\sum_{x_R}(\sum_{x_i} W_{x_R}^{\lambda_1} \chi_{x_i})} \\ \bar{V}_{\lambda_2}^{ht} = \frac{\sum_{x_R}(\sum_{x_i} W_{x_R}^{\lambda_2} \langle \bar{S}_{\lambda_2}\rangle_{x_i})}{\sum_{x_R}(\sum_{x_i} W_{x_R}^{\lambda_1} \chi_{x_i})} \\ W_{x_R}^{\lambda_1} = \frac{1}{\sigma_{\lambda_1}^2 N_{x_R}^{ht}} \\ W_{x_R}^{\lambda_2} = \frac{1}{\sigma_{\lambda_2}^2 N_{x_R}^{ht}} \tag{S-5}$$

where $x_R \epsilon X$, $x_i \epsilon \langle S_{\lambda_1} \rangle_{x_R}^{ht}$ or $\langle S_{\lambda_2} \rangle_{x_R}^{ht}$. χ_{x_i} is an indicator (0 or 1) of the interest domain. σ_{λ_1} and σ_{λ_2} are the standard deviations of the volumetric data and $N_{x_R}^{ht}$ is the number of non-zero coefficients of the corresponding 4D data after threshold truncation in the transformation domain.

Supplementary Text S3. Oxygen saturation (sO_2) computation via spectral unmixing

V_{λ_1} and V_{λ_2} are the volumetric data collected from the same sample. $\bar{V}_{\lambda_1}^{ht}$ and $\bar{V}_{\lambda_2}^{ht}$ are filtered volumetric data. $P(\lambda_1)$ and $P(\lambda_2)$ are maximum amplitude projections (MAP) of the $\bar{V}_{\lambda_1}^{ht}$ and $\bar{V}_{\lambda_2}^{ht}$. The molar extinction coefficient matrix is written as

$$\varepsilon = \begin{bmatrix} \varepsilon_{\text{Oxy-Hb}}(\lambda_1) & \varepsilon_{\text{Deoxy-Hb}}(\lambda_1) \\ \varepsilon_{\text{Oxy-Hb}}(\lambda_2) & \varepsilon_{\text{Deoxy-Hb}}(\lambda_2) \end{bmatrix} \tag{S-6}$$

where $\varepsilon_{\text{Oxy-Hb}}(\lambda_i)$ and $\varepsilon_{\text{Deoxy-Hb}}(\lambda_i)$ are the molar extinction coefficients of Oxy-Hb and Deoxy-Hb under different wavelengths (i = 1 or 2). Therefore, the molar concentrations of Oxy-Hb and Deoxy-Hb can be estimated by solving the following equation,

$$\varepsilon \begin{bmatrix} C_{\text{Oxy-Hb}} \\ C_{\text{Deoxy-Hb}} \end{bmatrix} = \begin{bmatrix} P(\lambda_1)/\phi_{\lambda_1} \\ P(\lambda_2)/\phi_{\lambda_2} \end{bmatrix} \tag{S-7}$$

where ϕ_{λ_1} and ϕ_{λ_2} are optical fluence under different wavelengths. The fluence changes are measured by a fast photodiode. The sO_2 can be determined from the following equation.

$$\begin{gathered} \begin{bmatrix} C_{\text{Oxy-Hb}} \\ C_{\text{Deoxy-Hb}} \end{bmatrix} = (\varepsilon^T \varepsilon)^{-1} \varepsilon^T \begin{bmatrix} P(\lambda_1)/\phi_{\lambda_1} \\ P(\lambda_2)/\phi_{\lambda_2} \end{bmatrix} \\ sO_2 = \frac{C_{\text{Oxy-Hb}}}{C_{\text{Deoxy-Hb}} + C_{\text{Oxy-Hb}}} \end{gathered} \tag{S-8}$$

Supplementary Text S4. Spatial resolution and imaging depth measurement

The lateral resolution of our system was measured by scanning the stainless-steel blade across the sharp edges. We fitted the amplitude of the photoacoustic signal across the sharp edge to obtain edge spread functions (ESF). Then the ESF function was derived to calculate the line spread functions (LSF). Finally, the full width at half maximum (FWHM) of the LSF was calculated for quantizing the lateral resolution. The axial resolution was measured by scanning a 10-μm-diameter tungsten filament. Then the A-line signals were extracted and the FWHM was calculated after a Hilbert transformation for quantizing the axial resolution.

To measure maximal imaging depth, we obliquely inserted a segment of a tungsten filament with a diameter of 250 μm into a fresh chicken breast tissue. The maximal imaging depth is determined by the 6-dB signal-to-noise ratio (SNR) compared with the background noise.

Supplementary Text S5. Maximum permissible exposure calculations for OR-PAM

The lateral resolution of OR-PAM is determined by the diffraction limits of optical spot size. We estimate the numerical aperture (NA) of the optical objective as follows,

$$R_{L,OR} = 0.51 \times \frac{\lambda}{NA} \tag{S-9}$$

where $R_{L,OR}$ is the experimentally measured lateral resolution (3.24 μm, Figure S3), and λ is the optical wavelength.

The maximum permissible exposure (MPE) is determined by several factors, such as spectral range and exposure duration on the tissue surface. Certain applications, for example, ophthalmic angiography, have more strict requirements (16). We calculate MPE on the tissue surface as follows,

The NA of the optical objective estimated from the equation (S-9) is 0.08. Because the wavelengths are between 400 nm and 700 nm, and the exposure duration T is less than 10 seconds, the MPE equation is written as

$$MPE = 1100 \times C_A \times T^{0.25} \ \ (mJ/cm^2) \tag{S-10}$$

where C_A is a wavelength correction factor and is unity for visible wavelength range. The calculated exposure duration T is written as,

$$T = \frac{2 \times D \times (0.51 \times \lambda)}{R_{L,OR} \times \Delta} \times \frac{1}{PRR} = N \times \frac{1}{PRR} \tag{S-11}$$

where D is an assumed depth of the optical focus and is estimated as 250 μm. Δ is the scanning step size and is 2.5 μm. PRR is the laser pulse repetition rate and is 4000 Hz here.

According to the equation (S-11), we know the exposure duration is 0.0042 seconds, and there are about N(16.75) adjacent laser pulses overlap on the skin surface. Combing (S-10) and (S-11), the MPE for the single wavelength on the overlapped region is 16.71 mJ/cm^2, and the maximal permissible single pulse energy is written,

$$MPE_{single} = \frac{MPE}{N} \times (\pi \times (\frac{D \times (0.51 \times \lambda)}{R_{L,OR}})^2) \tag{S-12}$$

According to the equation of (S-12), maximal permissible single pulse energy MPE_{single} is 230 nJ.

For sO_2 imaging, two wavelengths, 532 nm and 558 nm are used. Therefore, the PRR is doubled. However, the interval between the two wavelengths is only 300 ns and is a biologically negligible delay. The practical exposure duration is nearly kept unchanged, but because of the doubled pulse energy, the MPE_{single} reduces to half of its original value. If the energy of 532 nm and 558 nm is the same, the MPE_{single} is estimated as 115 nJ.
